# A Narrative Review on Shifting Practice and Policy Around Social Determinants of Health (SDOH) Screenings: Expanding the Role of Social Workers in Healthcare Settings in the U.S.

**DOI:** 10.3390/healthcare13101097

**Published:** 2025-05-08

**Authors:** Shetal Vohra-Gupta, Liana Petruzzi, Amulya Cherian, Cheng Chow, Monica Unzueta, Rachel Joachimi

**Affiliations:** 1 Steve Hicks School of Social Work, The University of Texas at Austin, Austin, TX 78705, USA; chengchow@utexas.edu; 2 Department of Health Studies, American University, Washington, DC 20016, USA; lpetruzzi@american.edu; 3 Analytics Department, Georgia Institute of Technology, Atlanta, GA 30332, USA; acherian@utexas.edu; 4 Asian Family Support Services of Austin, Austin, TX 78761, USA; munzueta@utexas.edu; 5 Austin Community Foundation, Austin, TX 78751, USA; rjjoachimi@gmail.com

**Keywords:** social determinants of health, social care interventions, healthcare, implementation science, primary care, social work roles

## Abstract

Introduction: Social workers play a critical role in healthcare settings by addressing both medical and nonmedical needs. Trained in human behavior and social environments, they are best suited to screen for social determinants of health (SDOH) and connect patients with resources paving the way for optimal health and well-being. Methods: This narrative review synthesizes the existing literature on SDOH screening practices within healthcare settings, emphasizing the role of social workers. A systematic search was conducted across multiple databases. A total of 26 studies met the inclusion criteria and were analyzed using a qualitative narrative synthesis approach. Results: This review reveals variability in SDOH screening domains, tools, and implementation strategies across healthcare settings. Facilitators and barriers to implementation were identified, including workflow integration, interprofessional collaboration, and contextual readiness. Social workers emerged as key professionals in addressing health-related social risks, leveraging their expertise in patient engagement, assessment, and system navigation. We further introduced the integrated-Promoting Action on Research Implementation in Health Services (i-PARIHS) framework to suggest the effective integration of SDOH screenings, emphasizing innovation, recipient engagement, contextual readiness, and facilitation. Conclusion: The effective integration of SDOH screenings requires structured workflows, interdisciplinary collaboration, and policy support. The review provides practice models of workflows for SDOH screenings and implications within two different healthcare settings: hospitals and outpatient clinics, offering insights into best practices and areas for future research. Strengthening the role of social workers in SDOH screenings can improve patient outcomes and promote health equity.

## 1. Introduction

The role of social workers in healthcare settings is critical to address both the medical and nonmedical needs of patients. While they are often recognized as case managers, care coordinators, and mental health providers, their capacity to address health-related social needs is equally essential. With biopsychosocial training and systems-based practice, social workers play a crucial role in identifying social risk factors, often referred to as social determinants of health (SDOH). Extensive research demonstrates that social, economic, and environmental factors influence health outcomes beyond clinical care alone [1,2]. Social workers also possess specialized knowledge to navigate complex healthcare systems and conduct comprehensive assessments, enabling them to connect patients to appropriate referrals and resources. Hospitals and primary care settings are particularly positioned to integrate SDOH screening, as they typically include social workers with training to address both the medical and nonmedical needs of patients.

Given that health inequities are often related to social risk, screening for SDOH is necessary to achieve health equity [3,4]. The data provided through SDOH screenings enable healthcare providers to identify, translate, and address risk factors, especially among marginalized populations [5]. Biopsychosocial assessments conducted by social workers may be considered a gold standard given the competencies and trainings required [3]. In the United States, the Council on Social Work Education (CSWE) outlines nine core competencies that guide social work practice and education. Competency 1 emphasizes ethical and professional behavior, while Competencies 2 through 5 focus on engaging diversity and difference in practice, advancing human rights and social justice, using practice-informed research and research-informed practice, and engaging in policy practice. Through specialized curriculum and clinical training, social workers are skilled at recognizing the implications of the larger social and economic context and how it affects the individual’s health and behavior (Competency 7). Additionally, they utilize evidence-based methods of engaging with clients or patients and understanding the importance of interprofessional collaboration within healthcare teams (Competencies 6 and 8). Competency 9 further underscores the role of social workers in evaluating practice effectiveness, making them well positioned to assess and refine SDOH screening methods. As a result, healthcare professionals have identified social workers as best suited to administer screenings and address health-related social needs within healthcare settings [6].

Social workers have long contributed to public health by addressing the conditions in which individuals are born, grow, live, work, and age. They bridge healthcare systems and community-based services, enhancing access and outcomes. Historically, social work and public health have shared a mission to reduce disparities and promote well-being, particularly among vulnerable populations [7,8]. Social workers not only provide direct services to individuals but also advocate for systemic changes that address the root causes of health inequities. For example, during the COVID-19 pandemic, social workers mobilized to support vulnerable groups across various settings, ensuring access to essential services, resources, and psychological support [9,10]. Their ability to work directly with individuals and families allows social workers to identify and address adverse social determinants such as poverty, housing instability, and limited access to healthcare resources [11,12].

## 2. Purpose and Background

This review was motivated by ongoing challenges in implementing SDOH screenings in healthcare settings, particularly around ensuring effectiveness, feasibility, and integration into routine care. While this work was initially informed by a small community-based project to validate a social risk screening tool at a clinic serving vulnerable populations in Central Texas, the broader aim is to examine how SDOH screenings are implemented and to explore their practice and policy implications. Several guiding questions emerged in the course of our inquiry:

- How are SDOH screenings being implemented in healthcare settings?
- Who is best to administer a SDOH tool in a healthcare setting?
- What are the gaps in the implementation of SDOH screenings?
- How to make SDOH screenings a regular part of patient care?

To address these questions, we identified relevant screening models currently in use, compared implementation workflows, and analyzed the role of social workers in SDOH screening processes [13]. The purpose of this review is to provide a comprehensive overview of existing SDOH screening practices and to highlight the current and potential roles of social workers in administering screenings and delivering social care interventions. We then introduce the integrated-Promoting Action on Research Implementation in Health Services (i-PARIHS), an implementation science framework of discovering barriers and facilitators to offer a suggested framework for implementing SDOH screenings within healthcare settings. Finally, we provide suggested workflows of when to best implement SDOH screenings during a patient’s visit.

## 3. Methods

### 3.1. Overview and Inclusion Criteria

This narrative review was conducted to reflect the interdisciplinary nature of healthcare and social care interventions, with a focus on the screening and implementation of SDOH assessments, the role of healthcare professionals (particularly social workers), and related workflows. To ensure a broad and comprehensive understanding of the topic, a narrative review approach was adopted. This methodology allowed us to synthesize pivotal papers and present a qualitative summary [14]. Given the purpose-driven nature of this review, inclusion criteria were intentionally focused on studies meeting the following conditions: (1) scope: studies that examined SDOH or social needs screenings in a healthcare setting; (2) implementation factors: articles discussing facilitators and barriers to the implementation of SDOH screening tools, including workflow integration; (3) professional roles: papers that explored the role of social workers or other healthcare professionals in conducting SDOH screenings. Exclusion criteria included articles outside the scope of healthcare settings, studies not addressing SDOH screenings, or papers lacking sufficient detail on implementation processes. Additionally, opinion pieces or editorials without empirical or theoretical support were excluded.

### 3.2. Search Strategy

The review process involved systematic searchers across seven databases to capture relevant studies published between January 2000 to December 2024. These included Academic Search Complete, APA PsycINFO, Education Source, ERIC, and SocINDEX. Also included were the PubMed (MEDLINE) and Web of Science electronic databases. Search terms were carefully selected to capture the breadth and depth of literature related to SDOH screening tools, their implementation in healthcare settings, and the role of healthcare professionals. Search terms included [“Social determinants of health” OR SDOH OR “social needs” OR “health-related social needs” OR “social risk” OR “health-related social risk”] AND [“screening” OR “assessment” OR “tool”] AND [“role of social worker” OR “role of healthcare professional”]. A secondary search string included terms to focus on implementation and workflow: [“Social determinants of health” OR SDOH] OR [“social needs” OR “health-related social needs” OR “social risk” OR “health-related social risk”] AND [“screening” OR “assessment” OR “tool”] AND [implementation] AND [workflow].

### 3.3. Data Extraction and Synthesis

The final set of included studies was reviewed independently by multiple authors to extract key themes related to SDOH implementation, screening tools, healthcare roles, and barriers. We used a thematic narrative synthesis approach to organize findings across these domains. Discrepancies in interpretation were discussed among team members until consensus was reached. This process allowed us to qualitatively integrate practical insights and identify recurring patterns relevant to social work practice and implementation science.

While the inclusion criteria were purposefully defined to align with the study aims, we acknowledge the potential limitations in generalizability. Nonetheless, this focused approach ensures the findings are directly applicable to healthcare contexts seeking to expand and refine SDOH screening workflows.

## 4. Results

**Figure 1:**
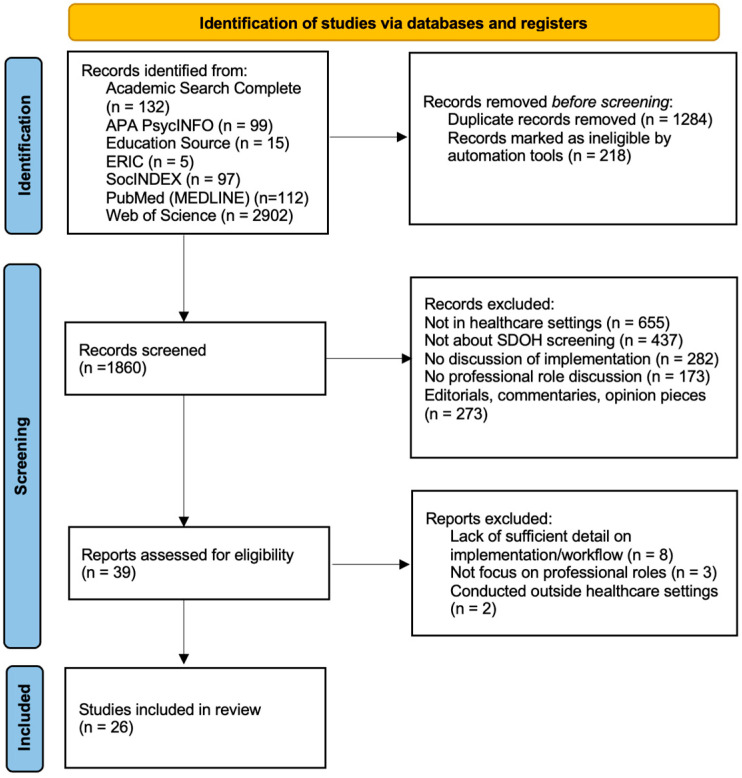
A flow diagram of the literature search process.

This work reports on understanding how SDOH screenings are currently used in healthcare clinics, the role of social workers in administering SDOH and barriers to implementation of SDOH screenings in clinical settings. While the importance of addressing SDOH is well documented, the screening of SDOH is lacking in effective and efficient implementation. The initial search yielded 3362 articles (see Figure 1 for the PRISMA flow diagram of the selection process). After applying criteria and screening the initial set of articles, a total of 26 studies were included in the review.

We identified role ambiguity, inefficiencies in SDOH screening implementation, variability in screening practices, and barriers to coordinating resources as significant challenges to effective implementation in clinical settings. Although this narrative review was specific to the project mentioned above, the findings support the need for discovering innovative models to integrate SDOH screening into all clinical settings. Table 1 presents a detailed summary of reviewed articles.

### 4.1. SDOH Screenings Being Used in Healthcare Settings

*SDOH screenings in primary care*. Screening for SDOH has been on the rise in primary care settings, as evidenced by one paper that describes 6 primary care clinics as cases for SDOH development and implementation [13]. However, only two of the six case studies identified who was assisting with the SDOH screening and resource referral, neither of which were social workers. In fact, while several review papers have been written about social workers in primary care settings, very few studies have described the role of social workers in screening for SDOH in primary care [38,39]. This creates challenges for researchers and practitioners alike in knowing to what extent and in what capacities social workers are involved in SDOH screenings. In the few studies that do explicitly identify the role of social workers in the SDOH screening workflow, social workers almost exclusively receive referrals within a community-based organization after a SDOH screening has been completed by another healthcare professional [21,34]. Further, a 2022 study shows that healthcare professionals identify social workers as the most appropriate profession to administer the screenings and connect patients to community resources directly [6]. However, only 2% agreed to a social work referral via an outside organization [34]. This warrants further exploration, as it may be due to barriers in attending a follow-up appointment, or unfamiliarity with a social worker’s role.

*SDOH screenings in hospital settings*. Standardized screening for SDOH in hospital systems is also on the rise through national programs such as the Accountable Health Communities [15,27]. A recent study identified social workers as an ideal workforce to assess and address SDOH, yet the role that hospital social workers currently play in this process is unclear [33]. Most studies that do report hospital social workers assessing or addressing SDOH are in pediatric hospital settings or outside the United States [21,31,40]. The only hospital-based study in the United States found that 23% of US level 2 to 4 NICUs have implemented a standardized screening of SD, and the process is primarily led by social workers (92%) during the first week of hospitalization [20]. The social workers reported utilizing standardized screenings including iHELP, Accountable Health Communities, PRAPARE, and SEEK; however, 35% reported using a non-standardized assessment created by their institution.

Two additional studies that discussed the role of social workers in assessing and addressing SDOH were based in Canada. The first study found that SDOH were a top priority for hospital social workers in Toronto, and over 90% of their time involved intervening on at least one SDOH [21]. Another Canadian study in a pediatric hospital reported that social determinants of health contributed to psychosocial complexity, with 30% reporting housing instability and 23% reporting food insecurity [30]. This study also tracked the top interventions provided by social workers, but social determinants of health were not included as an intervention. Social workers did report conducting assessments and providing of resources as two separate interventions, but both tasks were reported for less than 10% of their caseload. However, these findings may not be generalizable to the United States due to different healthcare policy contexts. Further research to close the practice gaps is needed to better understand and ultimately expand the role of hospital social workers in assessing and addressing SDOH.

SDOH screenings in other settings. We also want to review the work of healthcare social workers outside hospital and primary care settings that may be assessing and addressing SDOH. For example, social workers have been integrated into oncology and palliative care spaces since the late 1970s and patient navigation is often provided to oncology patients by social workers [19,41]. Patient navigation is defined as individualized assistance to help overcome healthcare system barriers and facilitate timely access to health services across the cancer care experience [42]. Due to the variety of professional backgrounds of patient navigators, there has been some formal delineation of roles within oncology patient navigation [25]. Further, an Association of Oncology Social Workers has existed since the late 1970s, which has recently released competencies for oncology social workers, with a focus on SDOH [36]. Therefore, social workers have been assessing and addressing SDOH in oncology settings for several decades.

There is consensus among oncologists that SDOH have a significant impact on cancer disparities. A recent survey of oncologists reported that 93% agreed that SDOH had a significant impact on their patient’s health outcomes [4,37]. While there is limited literature on the role of social work in formally assessing and addressing SDOH in oncology settings, one recent conference presentation found that a standardized SDOH screening in an oncology clinic led to a significant increase in social work referrals [22]. Another study piloted an interdisciplinary pilot program for breast cancer patients and found that almost 50% of patients had financial needs [43]. While more research is needed that further evaluates the efficacy of oncology social workers at improving care through addressing SDOH, it remains clear that SDOH screenings should be included as part of routine patient care. Understanding and addressing social risk and needs for patients is tied to overall healthcare outcomes. In the following section, we propose an implementation science framework to inform the systemic and successful implementation of SDOH screenings into patient care.

### 4.2. The Role of Social Workers in SDOH Screenings

While social workers have been assessing and addressing SDOH for decades, there is a dearth of literature on social work’s role and expertise in social care interventions or nonmedical needs, which assess and address the key health-related social needs impacting individuals and communities [44]. With the expansion of integrated behavioral health (IBH) programs, social work presence has increased in primary and specialty care clinics. However, it is unclear how often social workers are assessing or addressing SDOH in that role [38,39]. It is also worth noting that research suggests physicians do not have the training, resources, or time to assess or address SDOH [45,46]. Moreover, a recent study of healthcare professionals in an academic medical center identified social workers as the best positioned to screen for SDOH, and social workers reported significantly less barriers to resource referrals [6].

Despite widespread recognition of social workers’ expertise in addressing social needs, their involvement in SDOH screening remains inconsistent. Contributing factors include role ambiguity within clinical teams [6,34], limited institutional policies mandating social work-led screening [13,33], and insufficient reimbursement structures that often prioritize physician or nursing staff for screening responsibilities [47]. These systemic barriers highlight the need for stronger integration policies and clearer delineation of roles.

### 4.3. Challenges to Effective Implementation of SDOH Screenings

*Variability in screening tools*. A recent literature review on SDOH screenings highlights the variability across screening and limited validation or standardization [3]. Most SDOH assessments include the 11 social and behavioral Institute of Medicine (IOM) domains such as social connection, education, neighborhood conditions, and financial or resource strain [48,49]. However, some screenings include additional measures that are not included in the IOM domains. For instance, the Health Leads Social Needs Assessment includes questions about child-care, employment, and transportation [50]. And the PRAPARE (Protocol for Responding to and Assessing Patient Assets, Risks and Experience) tool includes questions about housing, employment, and legal issues. This variation in SDOH domains and questions makes it challenging to aggregate SDOH data across settings or easily compare local, state, or national data.

*Screening modalities*. The method of administering SDOH screenings poses another challenge. Screening modalities can include face-to-face, on paper, and electronically, which includes integration into electronic health records (EHRs) or software platforms like Healthify or HealthSteps, with all having their own pros and cons [2,24,50]. For example, while electronic administration may improve patient comfort when reporting sensitive information, such as household violence or substance abuse, reliance on technology can exclude patients with limited digital literacy or access to electronic devices.

*Role ambiguity and workforce challenges*. Social workers are frequently identified as the ideal professionals to administer SDOH screenings and address social needs due to their training and expertise [6]. However, the role of social workers in SDOH screening workflows remains inconsistently defined. Studies suggest that social workers often receive referrals only after other healthcare professionals conduct screenings, limiting their ability to comprehensively assess and address patients’ needs [34]. Furthermore, physicians and other healthcare providers frequently lack the time, training, or resources to assess or address SDOH [45], creating a gap in ownership of this critical aspect of patient care.

*Barriers to coordinating resources*. Our overview highlights that social workers are among those on the healthcare team who often administer these screenings. In addition, social workers provide a myriad of services upon a positive SDOH screening, such as connecting patients with resources or treatment, and enrolling patients in social service programs. They may also coordinate patient care by organizing meetings with the patient and the patient’s care team to form a care plan and achieve the patient’s goals [28]. Resources provided to patients after administering a SDOH screenings include enrollment in state benefits or getting connected to community-based resources, depending on the patient’s needs [50]. Additionally, hospital social workers often assist patients with transitioning to different levels of care, whether that is more acute (skilled nursing facilities) or less acute (outpatient care). It is essential for hospital social workers to identify any social needs at discharge as unaddressed social needs can lead to preventable hospital readmissions.

## 5. Discussion

### 5.1. Overview of Social Care Interventions: Addressing SDOH

The integration of addressing social care with medical care is crucial for vulnerable populations as they navigate systemic oppression as well as chronic illnesses, mental health needs, and disabilities. Connecting patients with services such as transportation, housing and financial support, food sources or other social services can significantly reduce healthcare costs by preventing hospital and emergency-room readmissions, which may improve health outcomes through increasing adherence to treatment. However, the largest national study to date found that two-thirds of participants had unresolved social needs at the end of healthcare navigation, and navigation did not increase access to community resources, most likely due to a shortage of community services available [16].

Screening for SDOH in and of itself is not a social care intervention. Rather, social care interventions require assistance with identifying, contacting, or navigating healthcare and social service resources. As a critical component of patient care, social care interventions assess and address SDOH, which significantly impacts individual health outcomes. A recent systematic review of social care interventions identified over 50 studies that addressed patient social and economic needs [51]. Most papers focused on the process and about less than one-third of papers included healthcare utilization outcomes. Generally, social care interventions involve a multidisciplinary team approach, yet a critical gap includes lack of ownership over addressing SDOH needs or nonmedical needs of patients. While there is limited information, most social care interventions currently take place in hospital or primary care settings, both of which generally hire social workers as case managers, behavioral health specialists, or both [40,52]. Considering social work expertise in conducting comprehensive, biopsychosocial assessments that include social needs, intimate knowledge of common healthcare barriers, and available healthcare and social service resources, they are a crucial workforce for not only conducting SDOH screenings but administering social care interventions as well. As the previous sections suggest, there is ample literature on the integration of SDOH screenings into healthcare settings. However, the majority of the information is focused on describing the social needs of the patient population as opposed to the implementation of SDOH screening, barriers, or facilitators.

### 5.2. Barriers and Facilitators in Implementation of SDOH Screenings

Studies show that SDOH screenings face significant implementation gaps, negatively impacting their effectiveness within healthcare settings. One key issue is the lack of standardized best practices for SDOH screening processes, highlighting the need for strategic goals, guidelines, and protocols to ensure consistency across different healthcare settings [34,52]. While several validated SDOH screening tools exist and are recommended for clinical use, the challenge lies in standardizing the collection of individual-level SDOH data and effectively integrating them into electronic medical records [53]. It is essential to incorporate regular, consistent SDOH screenings for all patients, not just those with complex needs, in both in-patient and out-patient clinics. However, barriers to implementation include time management and a lack of resources, mainly training and staff support [32,34]. Moreover, the burden placed on healthcare providers to conduct screenings without adequate support or compensation is a significant challenge that needs to be addressed to prevent provider burnout and ensure quality of care [26]. Developing and implementing quality measures for SDOH screenings are crucial to support the effectiveness of these programs and ensure that patients’ social risk factors are adequately addressed [17]. Another barrier to implementation is the mixed perceptions regarding which health professionals (nursing, social work, community health workers, clinicians, etc.) are best suited to have ownership over the development and implementation of the screening process [32]. We also know that healthcare professionals burnout and are overwhelmed by documentation requirements, so it can be complicated to add another screening to their workflow, especially one that requires additional follow-up [54]. Further, SDOH follow-up can be complicated and time consuming as it requires up-to-date knowledge of eligibility criteria for social services as well as community resources.

Implementation facilitators included support from institutional leadership, aligning electronic health record (EHR) features and workflows to minimize time, and establishing a resource list and relationship with local social services [18,23]. Other facilitators include sharing information with frontline practice managers and staff weekly and regularly evaluating and incorporating feedback. Overall, understanding the relationship between context dependency and organizational readiness may be critical to success in primary care settings. Addressing the gaps in implementing SDOH screening in healthcare settings requires a comprehensive approach that considers standardizing best practices, providing support for healthcare providers, developing quality measures, and addressing systemic issues that impact implementation.

## 6. Implementation of Screening and Workflow

### 6.1. Implementation Framework for SDOH Screenings for Social Workers

To improve the implementation of SDOH screenings, we turn to the field of implementation science, which focuses on methods and strategies to promote the adoption and integration of evidence-based interventions into healthcare. The i-PARIHS framework is suggested for implementing SDOH assessments and social care interventions in current healthcare settings. We recommend utilizing the expertise of social workers to develop better screening tools, practices, and more effective workflows.

#### Using (i-PARIHS) Framework to Implement SDOH Screenings

The i-PARIHS framework is a conceptual model developed to facilitate implementation of evidence-based research into healthcare practice [55]. This framework outlines the critical success factors of implementation which include four interacting core elements: context, innovation, facilitation, and recipients. Innovation refers to the evidence or intervention being implemented and involves robust scientific evidence as well as clinical expertise and patient preferences, acknowledging the multifaceted nature of what constitutes reliable evidence in healthcare [56]. Recipients refers to the individuals and groups who are affected by and responsible for the implementation such as patients, and healthcare professionals and healthcare leadership. Context refers to the environment in which the implementation will occur such as organizational culture, leadership support, and evaluation process. Facilitation refers to the guiding implementation strategy such as education, quality improvement, and the roles, skills, and attributes of facilitators.

**Figure 2:**
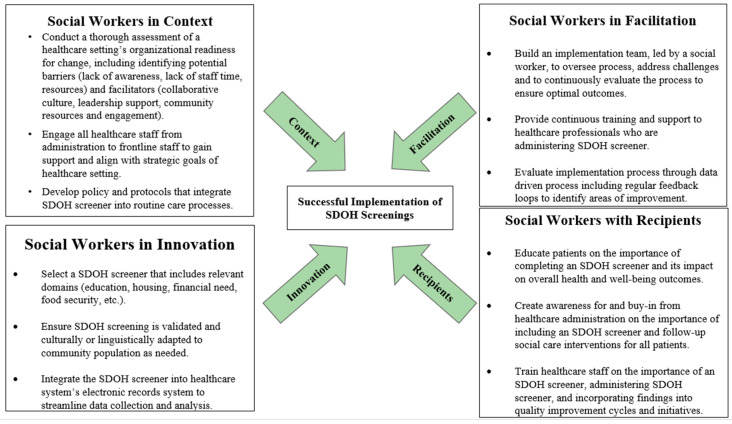
Integrated Promoting Action on Research Implementation in Health Services (i-PARIHS) framework illustating successful implentation of SDOH screenings by social workers in healthcare settings.

The i-PARIHS framework can serve as an ideal model for integrating social workers more effectively into healthcare settings, particularly with implementing SDOH screenings and follow-up care. Given the role social workers already play in a healthcare setting combined with competencies mentioned earlier, they bring the evidence-based tools and methods to conduct SDOH assessments. This, along with their expertise in patient and family engagement, their interaction with other healthcare team members, and knowledge of navigating organizational culture, positions them well to implement the most optimal process for implementing SDOH screening. In addition, social workers are familiar with community resources which ensures patients and their families who face gaps in social needs are efficiently addressed. We share an example of how social workers can implement the i-PARIHS framework in Figure 2.

- *Context*, as understanding and addressing the context within a healthcare setting is crucial for successful implementation.
- *Innovation*, implementing a SDOH screener which should be evidence-based and community friendly, with follow-up resources.
- *Facilitation*, with best practices to enable and sustain the implementation of a SDOH screener and follow-up resources.
- *Recipients*, which include patients, healthcare staff, and the administration.

Leveraging the i-PARIHS framework’s core elements—innovation, recipients, context, and facilitation—social workers can optimize the process of SDOH screening, ensuring it is evidence-based, culturally adapted, and seamlessly integrated into healthcare systems. This integration aligns well with our proposed workflows for SDOH screening in hospital and primary care settings, as it emphasizes the importance of a structured approach to identifying and addressing social needs throughout a patient’s healthcare journey.

**Figure 3:**
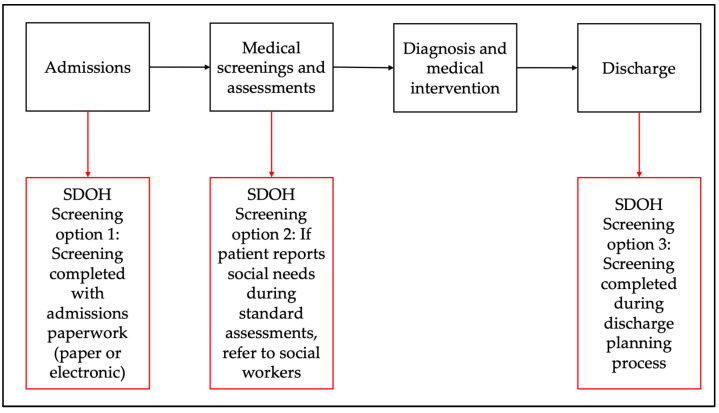
Proposed workflow for SDOH screening in hospital settings.

**Figure 4:**
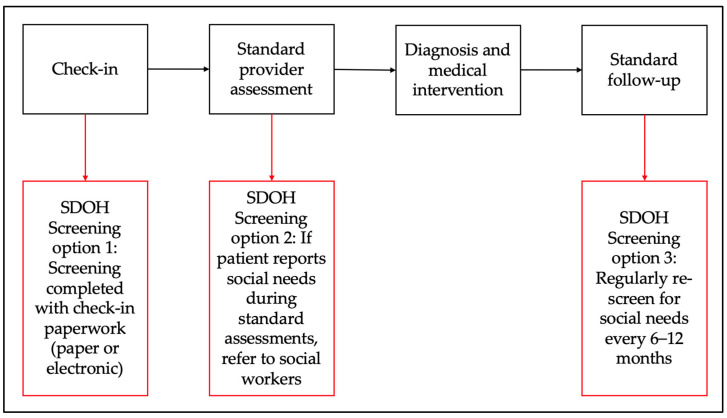
Proposed outpatient SDOH screening workflow.

Standard SDOH workflows typically begin with admission paperwork, but the process and personnel involved can vary. Some studies utilize medical assistants or front-desk staff for face-to-face administration, while others incorporate an electronic form into the admission process [26]. Social workers may be involved in identifying resources or following up with patients about health-related social risks where integrated. Based on the available literature, we have created two suggested workflows for SDOH screening in hospital and primary care settings to identify social risks. These workflows include three main points for screening: universal screening during admission or check-in, screening during the actual admission, or a provider visit with referrals to social work for identified risks, and screening during the discharge process in hospitals or during follow-up appointments in outpatient settings. (See Figure 3 and Figure 4).

In these workflows, electronic screening is preferred, as patients are more likely to report social needs and it requires less assistance from frontline staff [57]. This method can also generate a list of community resources based on social needs and zip code. During admission or provider visits, identified social needs can lead to referrals to social workers for appropriate interventions, such as enrollment in assistance programs. However, outpatient clinics may lack the workforce to address SDOH as effectively as hospitals, though medical assistants or Community Health Workers (CHWs) with proper training could fill this role. In hospitals, social needs reported during discharge planning can prompt SDOH screenings or direct referrals to resources, crucial for safe discharge. Incorporating SDOH screening into standard discharge practices ensures thorough support but may delay discharge due to time constraints on hospital-based social workers. Effective social care interventions involve tailored resource lists or structured interventions like case management, with follow-up being essential for success. Transitional care teams, where social workers follow up post-discharge, have shown efficacy in reducing hospital readmissions and can financially benefit hospital systems by avoiding Medicare penalties [58,59,60].

## 7. Limitations and Future Research

While this study highlights key aspects of SDOH screening implementation and social care interventions, several limitations should be acknowledged. First, most of the literature focuses on describing patient social needs rather than assessing the effectiveness of screening implementation across different settings. Future research should explore longitudinal outcomes, particularly how SDOH screenings influence healthcare utilization and long-term patient outcomes. Additionally, studies should evaluate the sustainability of SDOH interventions across diverse healthcare settings, including outpatient clinics and community-based care models. Future research should incorporate mixed-methods approaches, including longitudinal studies and experimental designs, to better assess the causal impact of SDOH screenings on healthcare outcomes.

Evaluating the effectiveness of SDOH screening models requires robust performance measures that assess both process outcomes (e.g., screening rates, provider adoption, and integration into workflows) and patient-centered outcomes (e.g., improved access to social services, reduced hospitalizations, and enhanced health outcomes). Common performance metrics include the proportion of patients screened for SDOH, referral completion rates, follow-up adherence, and the resolution of social needs. Additionally, patient-reported outcomes can provide insight into the impact of these interventions on overall well-being and healthcare utilization.

Implementation challenges also vary across care settings. In hospital settings, the focus is often on acute care needs, which can limit the time available for comprehensive SDOH screening and follow-up. Outpatient settings, on the other hand, allow for more continuous patient engagement, making them ideal for long-term social care interventions. To validate the effectiveness of SDOH screening models across these settings, comparative studies should examine differences in patient outcomes, resource utilization, and provider engagement. Standardized performance measures, such as tracking referral completion and follow-up success rates, will be essential in determining which models are most effective in addressing patients’ social needs. Future studies should further validate these models through standardized data collection across multiple healthcare systems to identify best practices.

## 8. Policy and Research Implications

There has been a major push to assess and address social determinants of health (SDOH) in healthcare settings since the creation of the Triple Aim and the passage of the Patient Protection and Affordable Care Act (ACA) in 2010 [61,62]. The Triple Aim focuses on (1) improving the patient care experience; (2) improving population health; and (3) reducing per capita healthcare costs [63]. As an outcome of Triple Aim, coordinated care and team-based care have been recognized as effective policy implementation strategies. In line with this, our narrative review shows a need to develop organizational policies that support improving the patient care experience, namely incorporating SDOH screenings within healthcare settings. Creating policy which implements SDOH screenings as part of every patient’s care eliminates gaps in social need and improves overall health and well-being. In addition, organizational policy around placing SDOH screenings under the prevue of social workers allows for competent and efficient assessments and the addressing of social needs. As mentioned above, policies around reimbursement for this work is also necessary. Recently, Medicare’s 2024 Physical Final Rule established that there would be no reimbursement for social workers for SDOH screenings or navigation, instead prioritizing a “qualifying billing practitioner” such as a physician [47]. Developing and applying concrete policies that define when, where, and how to administer SDOH screenings within a team-based structure work to improve patient care and eventually will positively influence population health for that community.

## 9. Conclusions

The integration of social workers to assess and address health-relevant social needs or the SDOH of patients in healthcare settings is essential to achieve optimal health outcomes for patients. However, implementation barriers such as a lack of standardized practices, time constraints, and workforce challenges must be addressed. Social workers, with their expertise in biopsychosocial assessments and knowledge of community resources, are well positioned to lead these efforts. The i-PARIHS framework provides a structured and evidence-based approach for integrating SDOH screenings into healthcare settings, ensuring their sustainability and effectiveness. By utilizing this framework, healthcare institutions can foster a systematic, team-based approach to screening and intervention, improving both individual patient care and broader health equity outcomes.

## Figures and Tables

**Table 1:** Reviewed articles.

No	Reference	Citation	SDOH Tool	Who	Screening Location	Implementation	Barriers or Takeaways
1	Alcaraz, Wiedt, Daniels, Yabroff, Guerra, and Wender, 2020	[4]	Accountable Health Communities (AHC) and PRAPARE tool	Patient navigators; healthcare providers	Community health centers, hospitals, payers, and public health departments	Integrated into electronic health records (EHRs) and assessed in both primary and specialist settings	Cross-sectoral partnerships and other coordinated approaches are required
2	Alley, Asomugha, Conway, and Sanghavi, 2016	[15]	Accountable Health Communities (AHC)	Community health workers	Community organizations, local health departments, managed-care organizations, clinical networks as well as clinical and community partners	Mobile device, in person at clinical sites, or through home visits depending on the setting	Gaps in item selection and variations in community needs and resources quality
3	Beil, Brower, and Chepaitis, 2023	[16]	Accountable Health Communities (AHC)	N/A	Participating bridge organizations	Hospitals for all Medicaid and Medicare beneficiaries	Gap to effective implementation includes community resource availability
4	Browne, McCurley, Fung, Levy, Clark, and Thorndike, 2021	[17]	11-item SDOH screening tool including food security, housing, transportation, cost-related medication underuse, paying for heat/electricity, child/eldercare, unemployment, and education	Program managers and resource staff	Community health centers	Patients completed the screening on an electronic tablet in the waiting room.	High caseloads, time constraints, inefficiencies in tracking, lack of community resources, and patient characteristics
5	Byhoff, Garg, Pellicer, Diaz, Yoon, Charns, and Drainoni, 2019	[18]	WE CARE SDOH screening tool	Medical assistants; provers and practice staff	Community health centers	(1) Distribution of a paper WE CARE screener by medical assistants; (2) medical assistants entered responses into the electronic medical record; (3) providers or practice staff printed resource information sheets for parents	No agreement on which domains to screen
6	Cantril and Haylock, 2013	[19]	National Comprehensive Cancer Care Program (NCCCP) Assessment Tool	Nurse navigators	N/A	N/A	N/A
7	Cordova-Ramos, Kerr, Heeren, Drainoni, Garg, and Parker, 2022	[20]	iHELP, AHC, PRAPARE, SEEK, or self-developed	Social workers	Hospitals	Administered screening tools to caregivers and/or in the first week hospitalization	Limited resources to implement a standardized tool, lack of adequate referrals to connect families in need, lack of validated screening tools
8	Craig, Bejan, and Muskat, 2013	[21]	A 12-item measure including healthcare services, housing, income and its distribution, social exclusion, gender, unemployment and employment security, aboriginal status, early life, disability status, education, employment and working conditions, and food security.	Health social workers	Hospitals	N/A	N/A
9	Crowe, 2022	[22]	A SDOH screening tool consists of eight domains	N/A	Rural cancer centers	Adult cancer patients completed a SDOH screening prior to their weekly oncology appointment	Lack of routine screening and documentation
10	Dauner and Loomer, 2021	[23]	Non-standardized screening	Care coordinators, case managers, primary care physicians, direct patient care professionals	Rural healthcare delivery organizations	N/A	No systematic screening process; Policies made it more difficult to use available resources and misaligned incentives between partners
11	Fleeger, Bottino, Boston Children’s Hospital, Hassan, Boston Children’s Hospital, Baker, Boston Public Health Commission, Kistler, Icahn School of Medicine at Mount Sinai, Pikcilingis, and ViiV Healthcare, 2016	[24]	HelpSteps.com, a web-based screening and referral system	Navigators; counselors; resident health advocates; Youth health ambassadors	Clinics, hospitals, community-based organizations	Bring tablets to the leadership meetings and resource fairs; use HelpSteps to find health and social services for residents in communities	Limited access to computers and the challenge of changing old habits
12	Franklin, Burke, Dean, Johnston, Nevidjon, and Simms Booth, 2022	[25]	Oncology Navigation Standards of Professional Practice	Professional navigators (nurse, social work navigators); community health workers	Clinics, hospitals, and communities	N/A	N/A
13	Gruß, Bunce, Davis, Dambrun, Cottrell, and Gold, 2021	[26]	EHR-based SDH screening informed by PRAPARE and AHC	Healthcare staff and professionals	Community health centers	Electronic health record (EHR)-based SDH screening	N/A
14	Highfield, Ferguson, Walsh, Paret, Ganelin, Hwang, and Morgan, 2020	[27]	AHC	CDS managers and staff	Clinical delivery sites (CDS)	N/A	Policy and procedures navigation; personnel turnover
15	Hostetter, Klein, and McCarthy, 2018	[28]	Vermont Self-Sufficiency Outcomes Matrix	Case managers; social workers; nurse; counselors	Care coordination agencies	Use diagrams to help patients and families visualize the social, financial, and medical support systems and ask them to select those that represent their immediate and longer-term priorities	N/A
16	Kostelanetz, Pettapiece-Phillips, Weems, Spalding, Roumie, Wilkins, and Kripalani, 2022	[6]	N/A	Physicians; social workers	Medical center	N/A	Lack of time, resources, standardized approaches, and professional burnout
17	LaForge, Gold, Cottrell, Bunce, Proser, Hollombe, Dambrun, Cohen, and Clark, 2018	[13]	Institute of Medicine (IOM) Recommended Patient-Reported SDH	N/A	Ambulatory care settings	Available in electronic health records (EHRs), patient portal, or on paper	Customization of recommended tools; need for flexibility
18	Meyer, Lerner, Phillips, and Zumwalt, 2020	[29]	AHC	Volunteers	Primary care practices	Screenings were completed on tablets via NowPow, a SDOH screening and referral platform, and were coupled with clinical screenings in the waiting room	Require the flexibility to change and adapt workflows; patients who seek care at these practices often feel overwhelmed by the healthcare system
19	Muskat, Craig, and Mathai, 2017	[30]	N/A	Hospital social workers	Pediatric hospital	N/A	N/A
20	Ponton, Courtemanche, Singh, Duffy, Courtemanche, and Loock, 2022	[31]	Surgery and Society Questionnaire (SASQ), Barriers to care, Economic factors, Adversity, Resiliency, and Social capital (BEARS)	Clinician; social workers	Hospital	Complete a paper copy of the questionnaire at check-in	N/A
21	Schickedanz, Hamity, Rogers, Sharp, and Jackson, 2019	[32]	Non-standardized social needs assessment	Physicians; social workers; nurses; pharmacists	Medical centers	Results indicated the social workers most suited to implement assessment	Lack of time to ask and resources to address social need
22	Schwartz, Herrmann, Librizzi, Gayle, Waloff, Walsh, Rucker, Herrera and Bhansali, 2020	[33]	Varied based on clinician/physicians	Hospitalists; nurses	Hospitals	Based on clinician/physician	Lack of time, resources, and a standardized inpatient screening tool
23	Sokol, Ammer, Stein, Trout, Mohammed, and Miller, 2021	[34]	Non-standardized social needs assessment	Practitioners; pediatricians	Pediatric clinics	Findings indicate a need for a well-established social work support team for effective implementation	Need for efficient workflow and established protocols to minimize time constraints, the value of screening all patients; identify optimal screening frequency and rationale; and communicate expectations for screening
24	Sokol, Mehdipanah, Bess, Mohammed, and Miller, 2021	[35]	Social needs questionnaire for pediatric patients	N/A	Care centers	Before a health maintenance pediatric examination, parents completed the screening	N/A
25	Zebrack, Doherty, Grignon, Guan, Miller, Nelson, Otis-Green, Rayton, Schapmire, and Wiener, 2024	[36]	Distress screening/management tools	Oncology social workers	Clinics where patient receiving cancer care	Oncology social workers	Reimbursement dependent on standardization on core set of skills
26	Zettler, Feinberg, Jeune-Smith, and Gajra, 201	[37]	No screening administered	Physicians (oncologists/hematologists)	N/A	Awareness was high to address SDOH; however, lack of screening and lack of defined roles of healthcare professionals to administer the screening	Limited time or resources to assist patients with social needs

## Data Availability

No dataset was used for this work.
